# Supplementary material for: A High-Performance Computing Implementation of Iterative Random Forest for the Creation of Predictive Expression Networks
Source: Genes (Basel). 2019 Dec 2;10(12):996. doi: 10.3390/genes10120996 (PMC6947651; doi:10.3390/genes10120996)
Supplement: Supplementary file 1 [file genes-10-00996-s001.pdf]

Supplementary Information

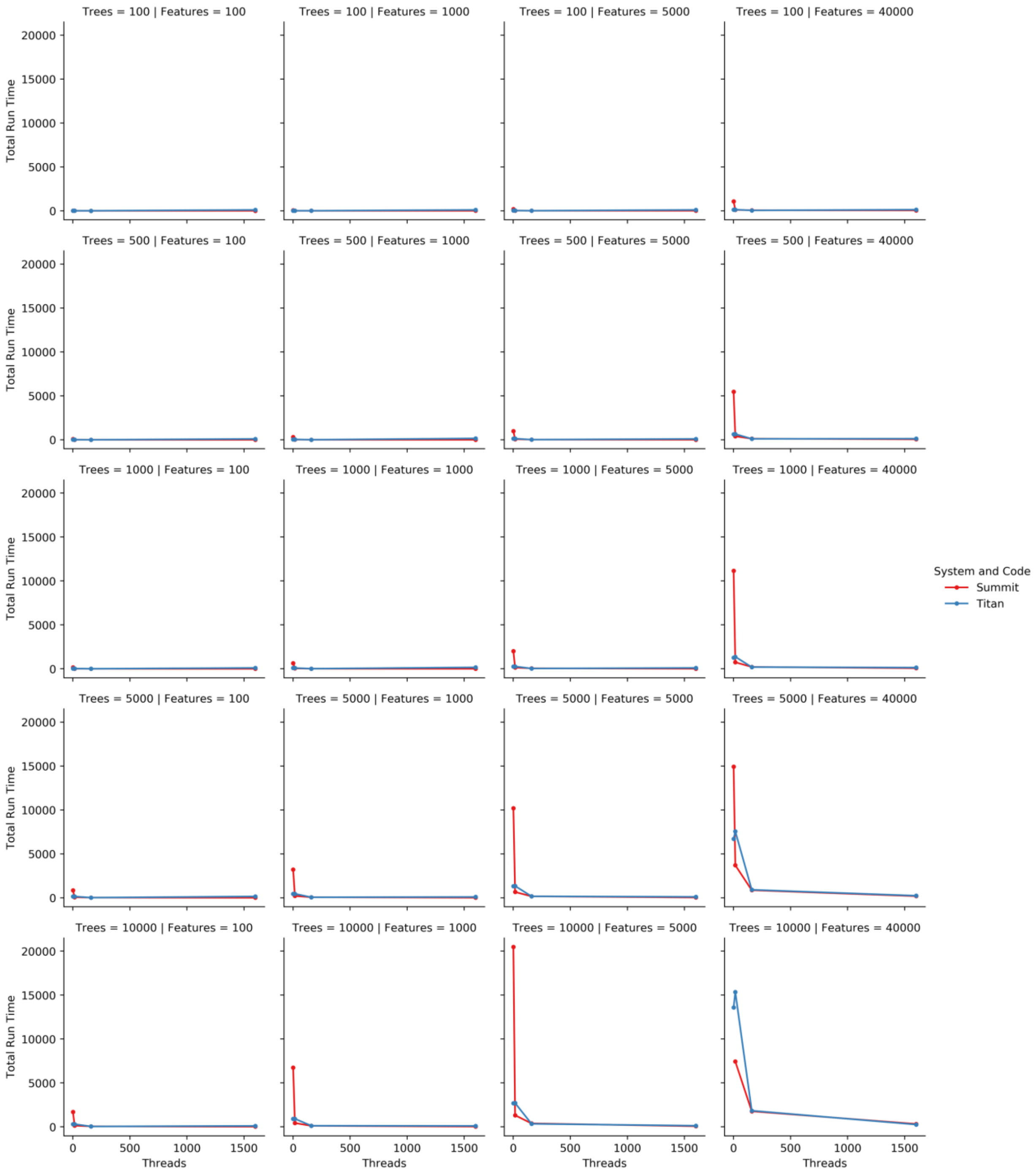

Figure S1. Runtime on HPC Systems. The full grid of graphs comparing Summit and Titan times to completion for the C++ code. The red line is for Summit and the blue line is for Titan.

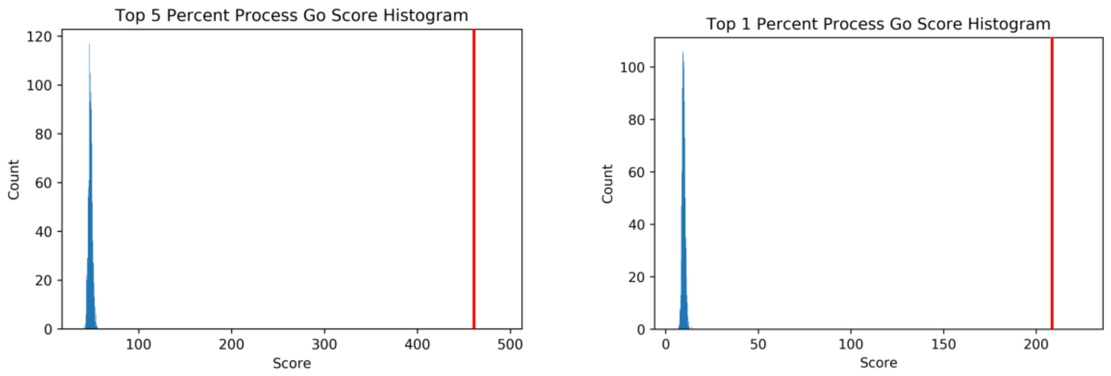

Figure S2. Null Distribution Plots. Null distrubtions with iRF intersect scores in red for the two thresholded Predictive Expression Networks not shown in the main text.

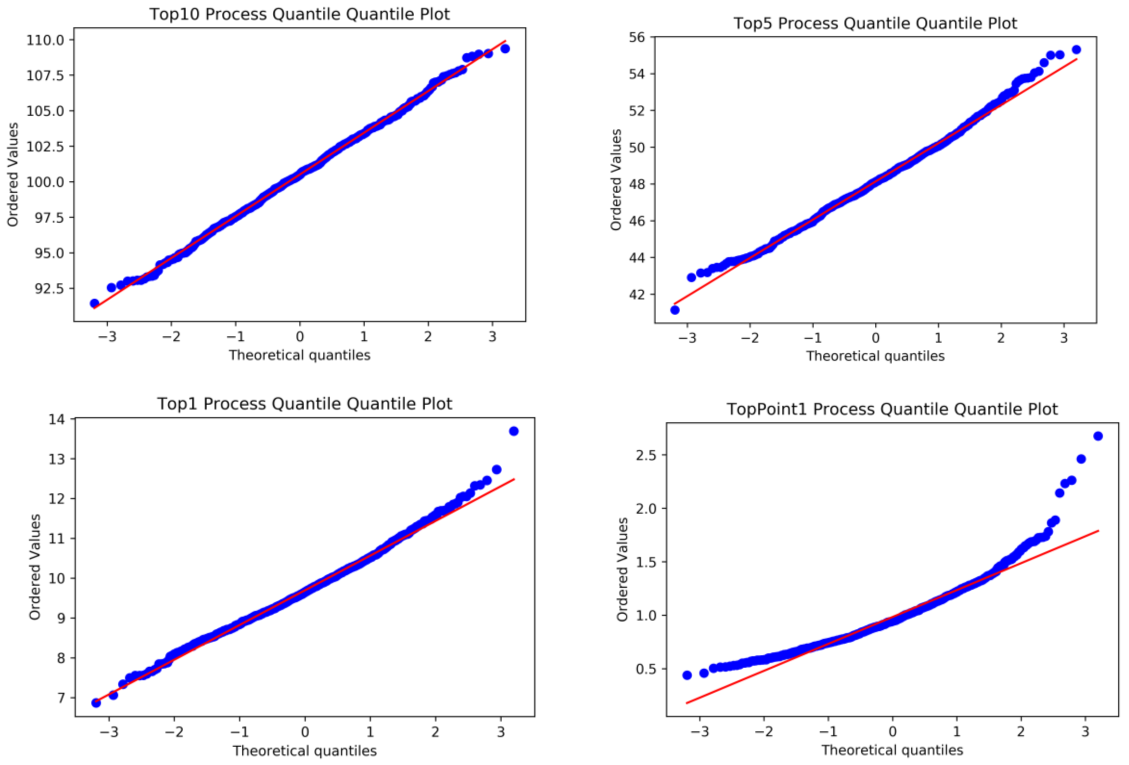

Figure S3. Quantile-Quantile Plots for the PENs. Quantile-quantile plots for each null distribution of the thresholded Predictive Expression Networks. .
